# A Pre-Formulation Study for Delivering Nucleic Acids as a Possible Gene Therapy Approach for Spinocerebellar Ataxia Disorders

**DOI:** 10.3390/molecules30173585

**Published:** 2025-09-02

**Authors:** Francesca Ferrara, Alfredo Sepe, Maddalena Sguizzato, Peggy Marconi, Rita Cortesi

**Affiliations:** 1 Department of Chemical, Pharmaceutical and Agricultural Sciences, University of Ferrara, 44121 Ferrara, Italy; frrfnc3@unife.it (F.F.); alfredo.sepe@edu.unife.it (A.S.); sgzmdl@unife.it (M.S.); 2 Biotechnology InterUniversity Consortium (C.I.B.), Ferrara Section, University of Ferrara, 44121 Ferrara, Italy; peggy.marconi@unife.it; 3 Department of Environmental and Prevention Sciences, University of Ferrara, 44121 Ferrara, Italy

**Keywords:** liposomes, gene delivery, spinocerebellar ataxias (SCAs) disorders, CRISPR/Cas9

## Abstract

Liposomes are lipid bilayer vesicles that are highly biocompatible, able to interact with the cell membrane, and able to release their cargo easily. The improvement of the physicochemical properties of liposomes, such as surface charge, lipid composition, and functionalization, makes these vesicles eligible delivery nanosystems for the gene therapy of many pathological conditions. In the present study, pre-formulation analysis was conducted to develop liposomes that facilitate the delivery of nucleic acids to neuronal cells, with the aim of future delivery of a CRISPR/Cas9 system designed to silence genes responsible for autosomal dominant neurodegenerative disorders. To this aim, different nucleic acid cargo models, including λ phage DNA, plasmid DNA, and mRNA encoding GFP, were considered. Liposomes with varying lipid compositions were produced using the ethanol injection method and analyzed for their dimensional stability and ability to interact with DNA. The selected formulations were tested in vitro using a neuroblastoma cell line (SH-SY5Y) to evaluate their potential toxicity and the ability to transfect cells with a DNA encoding the green fluorescent protein (pCMV-GFP). Among all formulations, the one containing phosphatidylcholine, phosphatidylethanolamine, pegylated 1,2-distearoyl-sn-glycero-3-phosphethanolamine, cholesterol, and dioctadecyl-dimethyl ammonium chloride (in the molar ratio 1:2:4:2:2) demonstrated the highest efficiency in mRNA delivery. Although this study was designed with the goal of ultimately enabling the delivery of a CRISPR/Cas9 system for treating autosomal dominant neurodegenerative disorders such as polyglutamine spinocerebellar ataxias (SCAs), CRISPR/Cas9 components were not delivered in the present work, and their application remains the objective of future investigations.

## 1. Introduction

Liposomes are nowadays considered pivotal nano-delivery systems due to their unique structural properties, as well as their biocompatibility and versatility in encapsulating various therapeutic agents. These nanosized vesicles, characterized by one or more phospholipid bilayers enclosing an aqueous core, are effective carriers for both hydrophilic and hydrophobic compounds [1,2]. Their amphiphilic nature enables favorable interactions with biological membranes, thereby facilitating efficient drug delivery across cellular barriers [3]. In recent years, liposomes have emerged as a promising non-viral delivery system for gene therapy, providing protection to sensitive nucleic acids from degradation and facilitating their delivery to target cells, thereby expanding their potential for clinical applications [4]. The efficacy of liposomes in gene delivery is primarily due to their biocompatibility and versatility of surface modifications. For instance, positively charged (cationic) liposomes have garnered significant attention due to their superior capacity to bind and deliver nucleic acids to cells while minimizing the cytotoxic effects often associated with alternative delivery methods [5,6]. Cationic lipids electrostatically interact with negatively charged nucleic acids, forming lipoplexes that promote nucleic acid cargo delivery across the cell membrane [7,8,9]. This interaction also enhances gene delivery by facilitating endosomal escape, a critical step for successful transfection in target cells [10,11]. Furthermore, advanced liposome modifications, such as incorporating specific targeting moieties or polyethylene glycol (PEG) chains, enhance the precision of delivery to selected tissues or cell types. These modifications also improve liposome stability and circulation time within the body, all crucial characteristics for applications in oncology and gene therapy [12,13].

Among cutting-edge genome editing technologies, CRISPR/Cas9 is now recognized as particularly promising because its ability to target specific genomic sequences can potentially permanently modify DNA, offering crucial strategies for the treatment of genetic diseases and disorders. Indeed, researchers utilize CRISPR/Cas9 and other gene editing techniques to correct mutations or regulate the expression of dysfunctional genes [14]. However, delivering large biomolecules, such as the CRISPR/Cas9 system, into cells presents significant challenges, primarily due to the need to cross the cell membrane barrier and mitigate off-target effects. In the present study, a pre-formulation analysis was conducted to develop liposomes for the potential delivery of a CRISPR/Cas9 system as a gene editing approach in the treatment of spinocerebellar ataxias (SCAs) [15]. Given the progressive nature of this heterogeneous group of neurodegenerative disorders caused by genetic mutations that affect neuronal function and survival, gene therapy emerges as a key approach for the treatment of SCA, especially because it has the potential to directly target the genetic defects responsible for these conditions. The integration of liposomes with the CRISPR/Cas9 system could overcome these issues by protecting the system’s components and efficiently delivering them into cells, enabling precise genome editing while minimizing systemic toxicity and off-target effects [16,17].

With this purpose, pre-formulating studies were conducted to identify liposomes with an optimal lipid composition that can carry nucleic acid cargo and promote its efficient delivery into cells. Liposomes with different lipid compositions, such as phosphatidylcholine (PC), cholesterol (CH), phosphatidylethanolamine (PE), pegylated 1,2-distearoyl-sn-glycero-3-phosphoethanolamine (DSPE-PEG2000), and the cationic lipid dioctadecyl-dimethyl ammonium chloride (DDAC), at different molar ratios, were produced by ethanol injection method and analyzed for their dimensional stability, and their ability to interact with nucleic acids cargo models, such as λ phage DNA or a plasmid encoding for the green fluorescent protein (pCMV-GFP). The in vitro study conducted on neuroblastoma cells (SH-SY5Y) showed that the selected liposomes were able to efficiently transfect cells either for the pCMV-GFP and an mRNA encoding GFP (eGFP), proving themselves to be good candidates for further studies investigating the delivery of a CRISPR/Cas9 system as possible gene therapy approach for SCA disorders.

## 2. Results and Discussion

### 2.1. Liposomes

Spinocerebellar ataxias (SCAs) represent a heterogeneous group of neurodegenerative disorders primarily characterized by progressive impairments in motor coordination caused by degeneration of the cerebellum and its associated neural pathways. More than 40 subtypes of SCAs have been identified, most of which follow an autosomal dominant inheritance pattern. The genetic causes frequently involve expansions of CAG repeats, which encode polyglutamine stretches, in genes such as ATXN1 (associated with SCA1) and ATXN3 (SCA3), as well as point mutations or other alterations in genes like CACNA1A (linked to SCA6). These genetic defects affect proteins essential for neuronal function and survival, leading to the progressive neurodegeneration observed in SCAs [15]. A previous study successfully used a CRISPR/Cas9 method with sgRNAs targeting Exon 8 of the ATXN1 gene to reduce both wild-type and mutated forms of ATXN1 protein. This silencing strategy substantially reduced ataxin1 expression with minimal off-target effects in fibroblasts from SCA1 patients, supporting CRISPR/Cas9 as a promising therapeutic approach for diseases with expanded polymorphisms [18]. Liposomal formulations are crucial for enhancing gene delivery because they help overcome challenges such as off-target effects, low transfection efficiency, and the limited bioavailability of the cargo. With this in mind, we conducted a pre-formulating study to find liposomal formulations that could enhance nucleic acid delivery. Moreover, the use of liposomes in a gene delivery approach can mitigate issues related to the use of transfection agents such as Lipofectamine, which can often promote cell toxicity and low transfection efficiency [19].

The ethanol injection method was chosen to obtain liposomes due to its ability to consistently produce smaller, more stable particles that offer superior transfection efficiency for gene delivery applications compared to other techniques [20,21]. The formulations were divided into three different groups based on their lipidic composition, as reported in Table 1.

#### 2.1.1. Group I Liposomes

To the first group of liposomes belong PC1, PCD1, and PCD2 (Table 1). PC1 is the simpler formulation being composed of PC:CH (4:1 mol/mol) and possesses a ζ-potential very close to neutrality (see Table 2); therefore, its electrostatic interaction with nucleic acid molecules is quite low [22,23]. To increase the charge of liposomes and thus favor the possible complexation with nucleic acids, the cationic surfactant DDAC was included in liposome composition at different molar ratios, obtaining PCD1 (PC:CH:DDAC 4:2:1 mol/mol/mol) and PCD2 (PC:CH:DDAC 4:2:1 mol/mol/mol) formulation reaching ζ-potential values higher than +65 mV (Table 2).

The research literature indicates that the use of DDAC enhances the liposome’s electrostatic properties and stability in biological environments. This leads to longer circulation times, better tissue delivery, and reduced cell toxicity compared to other cationic lipids, making DDAC a safer option for therapeutic use [24].

The formulations obtained were, in addition, evaluated in terms of size, and size distribution. As shown in Table 2**,** the formulations displayed vesicles with Z-average size < 135 nm and good polydispersion (PdI < 0.4), indicating the effectiveness of the ethanol injection method in obtaining homogeneous liposomes with a small size. As compared to PC, the presence of DDAC promoted a reduction in liposome size for either PCD1 or PCD2, and an increase in particle’s charge. Indeed, concerning the effect on size, the presence of a charged surfactant leads to a loss of positional correlations between vesicle bilayers probably due to the surface charge density conferred on the bilayers that results in a reduction in lamellarity and size. However, the transition from multi- to unilamellar vesicles is driven by the combination of some conditions, such as the system entropy, the surfactant content or temperature, and other terms [25,26,27,28].

On the basis of the vesicle characterization results, PCD2 was selected as a possible candidate for nucleic acid delivery and was therefore subjected to the formation of complexes with a DNA model, such as bacteriophage λDNA (lipoplex).

λDNA serves as an effective model system in gene delivery research due to its relatively simple composition and well-characterized genetic elements, which enable a more detailed investigation of the dynamics of DNA packaging, condensation, and transfection compared to larger or more complex DNA constructs [29,30]. For the evaluation of lipoplex formation, λDNA was incubated with PCD2 at concentrations from 40 to 80 µM for 15 min to allow interaction. Afterwards, an electrophoretic run on an agarose gel was conducted.

**Figure 1:**
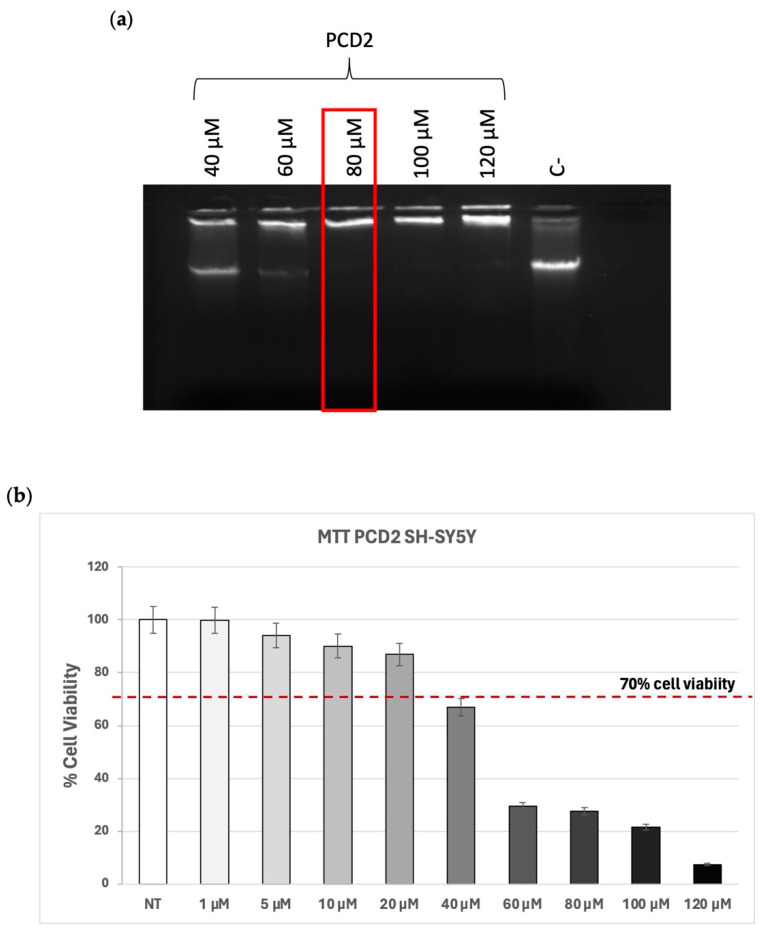
Group 1 formulation. (**a**) Complex formation between 1 µg of λDNA and PCD2 formulation at the indicated doses of DDAC. The red frame indicates the DDAC dose of liposomes at which the formation of the complex with the λDNA occurred. The negative control (C−) represents PCD2 liposome not incubated with λDNA; (**b**) MTT assay on SH-SY5Y cells treated with PCD2 formulation at the selected doses (1–120 µM) for 24 h. Cell viability is expressed as a percentage compared to non-treated (NT) cells. The accepted cell viability was considered to be 70%. Data are the mean of three different experiments + s.d.

As shown in Figure 1a, the ability of PCD2 to interact and complex with λDNA occurred starting from DDAC 80 µM. Indeed, when lipoplex formation occurs correctly, the dimensions become such that they prevent the migration of DNA through the agarose gel. To test the toxicity of PCD2, human glioblastoma cells (SH-SY5Y) were treated with different concentrations of PCD2, ranging from 1 to 120 µM, and an MTT assay was performed to evaluate cell viability after 24 h of treatment with the selected formulation. The data show that cells treated at a dose of 40 µM and above displayed a decreased viability (less than 70%) compared to the lower doses (Figure 1b). The in vitro cytotoxicity experiments enabled the definition of the optimal concentrations of cationic surfactant (10–20 µM), ensuring cell viability higher than 70%.

Considering that at the dose of DDAC 60 µM, the N/P ratio between the lipids and the nucleic cargo was very low (1.6/1) (see Table S1), it would seem that PCD2 formulation was not able to interact with the λDNA being the doses of DDAC lower than 80 µM. Indeed, the N/P ratio quantifies the balance of charges within a lipoplex. Specifically, it is the ratio of nitrogen (N) atoms found in the cationic lipid (carrying positive charges) to the phosphate (P) groups of the nucleic acid (DNA or RNA) (carrying negative charges). This ratio highlights the crucial balance between the positive charges from the lipid carrier and the negative charges from the nucleic acid, and it is, therefore, essential for the effective and safe delivery of nucleic acids via lipoplexes, impacting their encapsulation, cellular delivery, protection, cell viability, and ultimately, the success of gene therapies [31].

#### 2.1.2. Group II Liposomes

Given that the initial PCD2 formulation effectively complexed with λDNA only at a cell-toxic 80 µM DDAC dose, we optimized the liposome composition, aiming to achieve nucleic acid interaction with a reduced and safer DDAC concentration. As phosphatidylethanolamine (PE) is widely used to enhance nucleic acid delivery, we incorporated this phospholipid into liposome formulation at two different molar ratios obtaining the formulations PE1 and PE2 (see Table 1).

As a matter of fact, cationic liposomes featuring PE or its derivatives as neutral helper lipids result in increased cellular uptake and enhanced siRNA delivery compared to those solely composed of cationic lipids [32]. This synergistic effect is attributed to the ability of PE to facilitate membrane fusion and promote endosomal escape, thereby improving transfection rates [33]. In addition to other benefits, the incorporation of PE and its derivatives into cationic liposomes can reduce their cytotoxicity due to their ability to modulate membrane interactions and liposomal surface properties [34,35]. Indeed, cationic liposomes often exhibit increased cytotoxicity due to their positive surface charge, which causes destabilization of host cell membranes and induces reactive oxygen species (ROS) production [36,37].

As shown in Table 3, the mean size of PE1 formulation is larger (with a higher polydispersity index) than that of PE2, which displayed a Z-average particles diameter of around 145 nm and a homogeneous particle population (PdI = 0.172). For this reason, the PE1 formulation was subjected to extrusion, resulting in more homogeneous PE1 EXTR liposomes with a Z-average value of approximately 200 nm and a lower polydispersity index.

To test whether the formulations were suitable for nucleic acid cargo delivery, a plasmid encoding the green fluorescence protein GFP (pCMV-GFP) was used as a nucleic acid model to be transfected within neuronal-derived SH-SY5Y cells. The pCMV-GFP construct enables straightforward tracking of gene expression, as green fluorescent protein (GFP) serves as a robust fluorescent marker, allowing for both quantitative and qualitative assessments of transfection efficiency in various cell types [38,39].

This characteristic makes it particularly valuable in evaluating new gene delivery systems, as researchers can easily measure the intensity of GFP fluorescence to infer the levels of gene expression. Moreover, the versatility of the pCMV-GFP plasmid facilitates its integration into various experimental protocols, including those involving CRISPR/Cas9 and siRNA delivery systems. For example, Mao et al. explored targeted integration methods utilizing an eGFP knock-in model to assess gene editing success rates [40].

**Figure 2:**
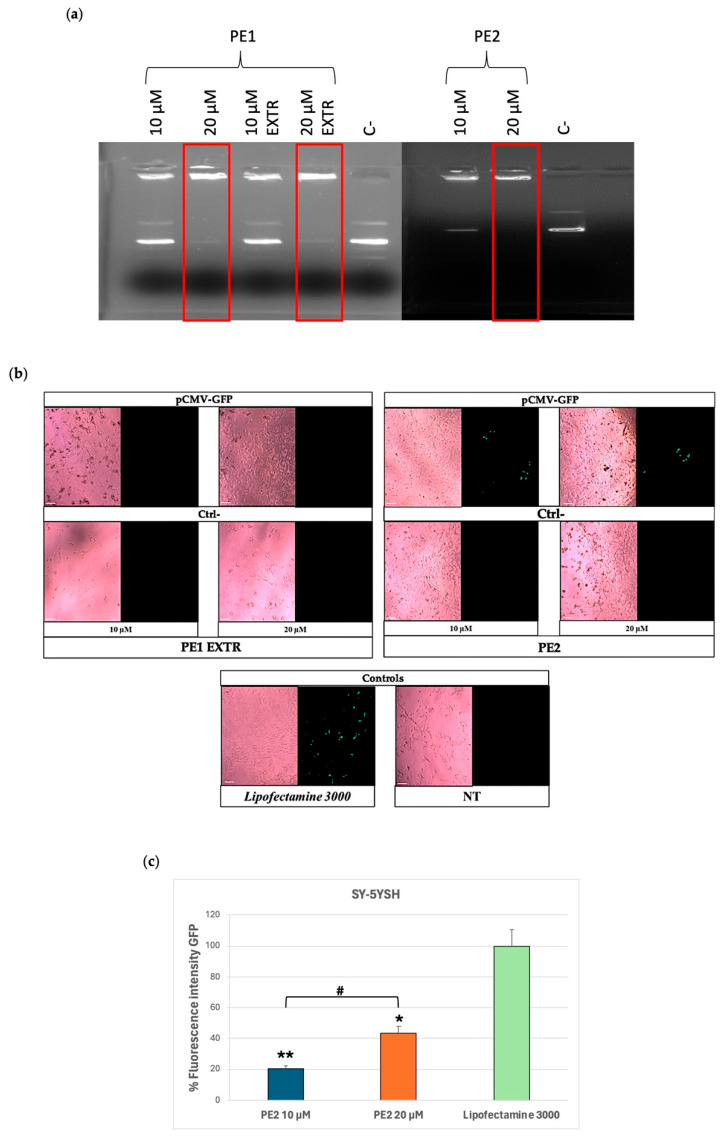
Group II formulations. (**a**) Complex formation between pCMV-GFP and PE1/PE2 formulations at the indicated doses of DDAC assessed by agarose gel electrophoresis. The red frames indicate the DDAC doses of liposomes at which the formation of the complex with the pCMV-GFP occurred. The negative control (C−) represents PE liposomes not incubated with pCMV-GFP. (**b**) Expression of GFP in SY-5YSH cells transfected with PE-pCMV-GFP lipoplexes at 10 and 20 µM of DDAC. The green staining represents GFP. Lipofectamine 3000 was used as a positive control, the negative controls (Ctrl−) are cells treated with the PE formulations without pCMV-GFP, whereas the not-treated (NT) cells represent cells left untreated. Images were taken at 10× magnification; scale bar is 200 µm. (**c**) Quantification of the GFP fluorescent signal in SY-5YSH cells transfected with PE2-pCMV-GFP lipoplexes by ImageJ V1.53 software. Data are the mean of three different experiments + s.d.; * *p* < 0.01 and ** *p* < 0.001 for PE2 10 µM or PE2 20 µM vs. Lipofectamine 3000; and # *p* < 0.01 as indicated by 2-way ANOVA followed by Tukey’s post hoc comparison test.

A complexation experiment was conducted to test whether the formulations could interact with the pCMV-GFP at the selected DDAC content of 10 µM and 20 µM. The electrophoretic run showed that either PE1 or PE2 formulations complex pCMV-GFP at 20 µM DDAC content, indicating the ability of the two formulations to interact more effectively with a nucleic acid cargo compared to the formulations of Group I (Figure 2a). Moreover, the extrusion of PE1 did not appear to affect its ability to complex DNA.

Of note, the N/P ratio of the lipoplex was much higher compared to PCD2 formulation, with a N/P ratio of 40/1 for 20 µM DDAC content and 20/1 for 10 µM DDAC content (Table S1), confirming that the change in lipidic composition allowed for a better complexation of pCMV-GFP.

For this reason, PE1 EXTR and PE2 formulations were then tested in vitro to verify their ability to transfect cells with pCMV-GFP at the selected doses of 10 and 20 µM. Cells were treated with the formulations for 48 h, and the GFP expression was evaluated by fluorescence microscopy. The results of the analysis showed that cells treated with PE1 EXTR were not transfected with GFP, as no fluorescence signal was detected compared to the positive control, Lipofectamine 3000 (Figure 2b), a widely used transfection reagent composed mainly of cationic lipids.

On the contrary, PE2 formulation showed a GFP signal of 40% compared to the positive control, indicating a promising role for this formulation in possible vehicle nucleic acid (Figure 2c). The low transfection efficiency of PE1 formulation (20% compared to Lipofectamine 3000) (Figure 2c) might be due to the extrusion process or to the presence of a lower concentration of PE in the liposome’s composition compared to PE2 formulation. Indeed, while extrusion is a common method for standardizing liposome size and often boosts transfection efficiency, its impact on transfection can be different. This is because extrusion’s effects are highly dependent on the lipid composition, extrusion membrane pore size, and the biophysical changes that result [41]. In our study, despite extruding the PE1 formulation to reduce size and polydispersity, and increasing the surface charge, we observed no difference in z-potential between PE1 and PE1 EXTR (Table 3). This resulted in lower transfection compared to the PE2 formulation, which exhibited a higher surface charge being +92 mV the z-potential. Furthermore, the increased PE concentration in PE2 likely contributed to its superior transfection rate since it favors cell membrane fluidity and interaction between liposomes and the cell membrane [35,42].

The results suggest that PE2 liposomes may be used for gene delivery, potentially overcoming the side effects of Lipofectamine, such as cytotoxicity. Indeed, while Lipofectamine remains a popular choice for nucleic acid delivery due to its proven efficiency, its associated cytotoxic effects necessitate a careful evaluation of the experimental design. The concentration of Lipofectamine used during transfection plays a crucial role in determining cytotoxicity since studies have shown that higher concentrations correlate with reduced cell viability, suggesting a dose-dependent toxicity [19,43]. While lower concentrations might enhance transfection efficiency, they may not sufficiently engage all cellular pathways necessary for successful gene delivery, prompting researchers to explore alternative non-viral vectors that can achieve comparable transfection rates with reduced toxicity.

#### 2.1.3. Group III Liposomes

Given the promising results obtained with the formulation of Group II, the liposome composition was modified to include DSPE-PEG. One significant benefit of incorporating DSPE into liposome formulations is its capacity to enhance their stability and increase their circulation time. DSPE-PEG conjugates, such as DSPE-PEG2000, have demonstrated a notable ability to prolong drug half-lives by hindering their rapid elimination from the bloodstream, thereby optimizing the pharmacokinetics and biodistribution of liposomal-delivered drugs [44,45]. This is primarily attributed to the stealth characteristic imparted by the PEG moiety, which reduces opsonization and recognition by the immune system, allowing liposomes to evade rapid clearance by the reticuloendothelial system (RES) [12]. Studies reveal that DSPE-PEG polymers lead to greater gene expression in neuronal cells, demonstrating their superior transfection efficiency compared to traditional PE-based liposome formulations [46,47].

Two different formulations with different concentrations of DSPE-PEG were developed, namely PED1 and PED2 (Table 1). Given their increased size, possibly due to the presence of DSPE, both formulations underwent extrusion allowing the obtaining of liposomes with reduced mean size (approximately 150 nm Z-average) and homogeneous population (PdI < 0.37) (see Table 4).

Moreover, PED1 showed higher *ζ*-potential values compared to PED2, probably due to the presence of a higher concentration of DSPE-PEG that may cause an increase in particle dimension and surface area.

**Figure 3:**
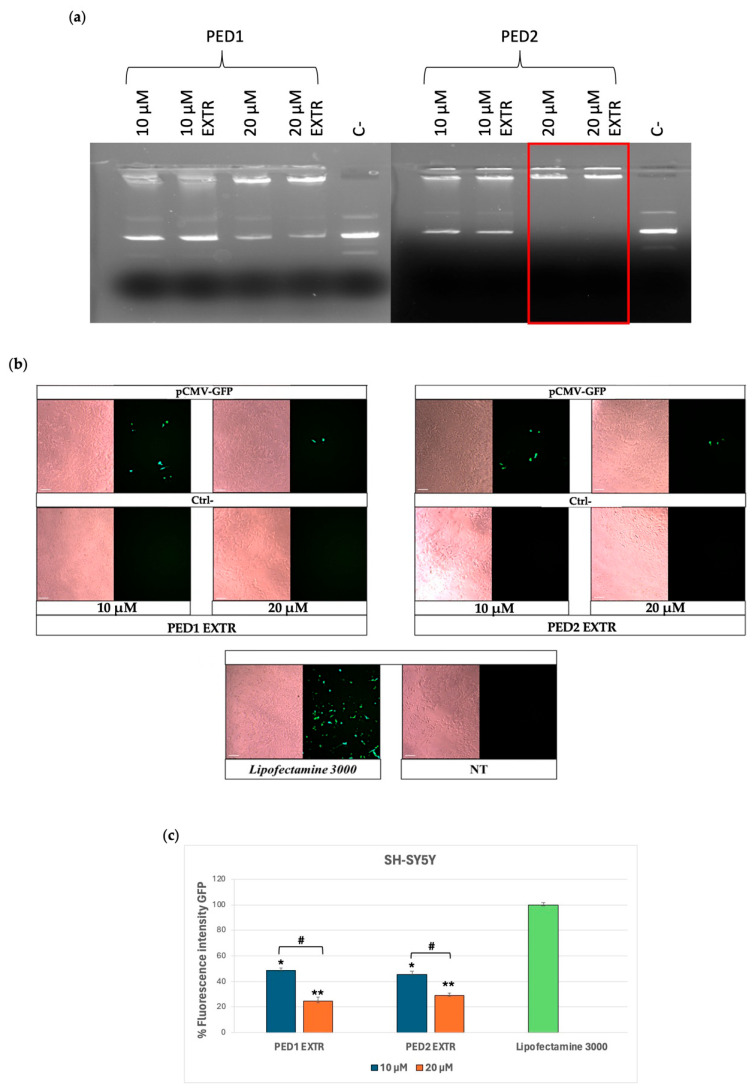
Group III formulations. (**a**) Complex formation between pCMV-GFP and PED EXTR formulations at the indicated doses of DDAC assessed by agarose gel electrophoresis. The red frames indicate the DDAC doses at which the formation of the complex occurred. The negative control (ctrl−) represents cells treated with the two formulations not carrying pCMV-GFP. (**b**) Transfection of SY-5YSH cells with PED EXTR formulations-pCMV-GFP lipoplex at 10 and 20 µM of DDAC. Lipofectamine 3000 was used as a positive control; the negative control (Ctrl−) represents cells treated with PED liposomes not carrying the pCMV-GFP, whereas the not-treated (NT) cells represent cells left untreated. Images were taken at 10× magnification; scale bar is 200 µm. (**c**) Quantification of the fluorescent expression levels of GFP in transfected SY-5YSH cells with PED liposomes by ImageJ V1.53 software. Data are the mean of three different experiments + s.d.; * *p* < 0.01 and ** *p* < 0.001 for PED1 EXTR 10 µM or PED2 EXTR 20 µM vs. Lipofectamine 3000; and # *p* < 0.01 as indicated by 2-way ANOVA followed by Tukey’s post hoc comparison test.

Despite the higher surface charge, the complexation experiment showed that PED1, either before or after extrusion, displays a slight tendency to associate with pCMV-GFP at both DDAC content (10 and 20 µM) (Figure 3a). On the other hand, PED2 liposomes displayed a more evident ability to complex pCMV-GFP at 10 µM of DDAC compared to the negative control and a stable complexation of pCMV-GFP at 20 µM of DDAC (Figure 3a). This behavior may be due to the higher concentration of PEG 2000 moiety within PED1 formulation, which may cause a steric hindrance, thus reducing the complexation of the plasmid. Indeed, although PEG-modified liposomes enhance stability and reduce non-specific interactions with biomolecules when used at a certain concentration, PEG chains can lower the adsorption of nucleic acids on nanoparticles [48]. Consequently, the length and concentration of PEG must be optimized to avoid hindering the delivery of nucleic acids via liposomes or reducing the transfection efficiency [49]. Nevertheless, both formulations maintained a high N/P ratio between lipids and the pCMV-GFP cargo (Table S1), indicating their suitability for the transfection study.

In particular, for the transfection experiment, PED1 EXTR and PED2 EXTR associated with pCMV-GFP were tested at DDAC concentrations of either 10 µM or 20 µM on SH-SY5Y cells. As shown in Figure 3b,c, the formulations in all conditions were able to transfect the cells efficiently. Even though the electrophoretic study showed better interaction between the formulations and pCMV-GFP at a 20 µM dose, PED formulations yielded higher GFP expression in cells when transfected at 10 µM, as depicted in the GFP fluorescence intensity graph in Figure 3c.

It is worth mentioning that the transfection efficiency of liposomes is highly dependent on the lipidic composition, the charge ratio of liposome/DNA complexes, as well as their size after extrusion [50]. Therefore, it might be possible that although the interaction with the plasmid was less evident at the 10 µM DDAC content, the presence of DSPE might have increased the transfection efficiency of liposomes even at a lower concentration of DDAC. Moreover, liposomes containing 10 µM of DDAC may be less toxic to the cells, thereby enhancing the number of cells that can be transfected compared to the 20 µM content.

**Figure 4:**
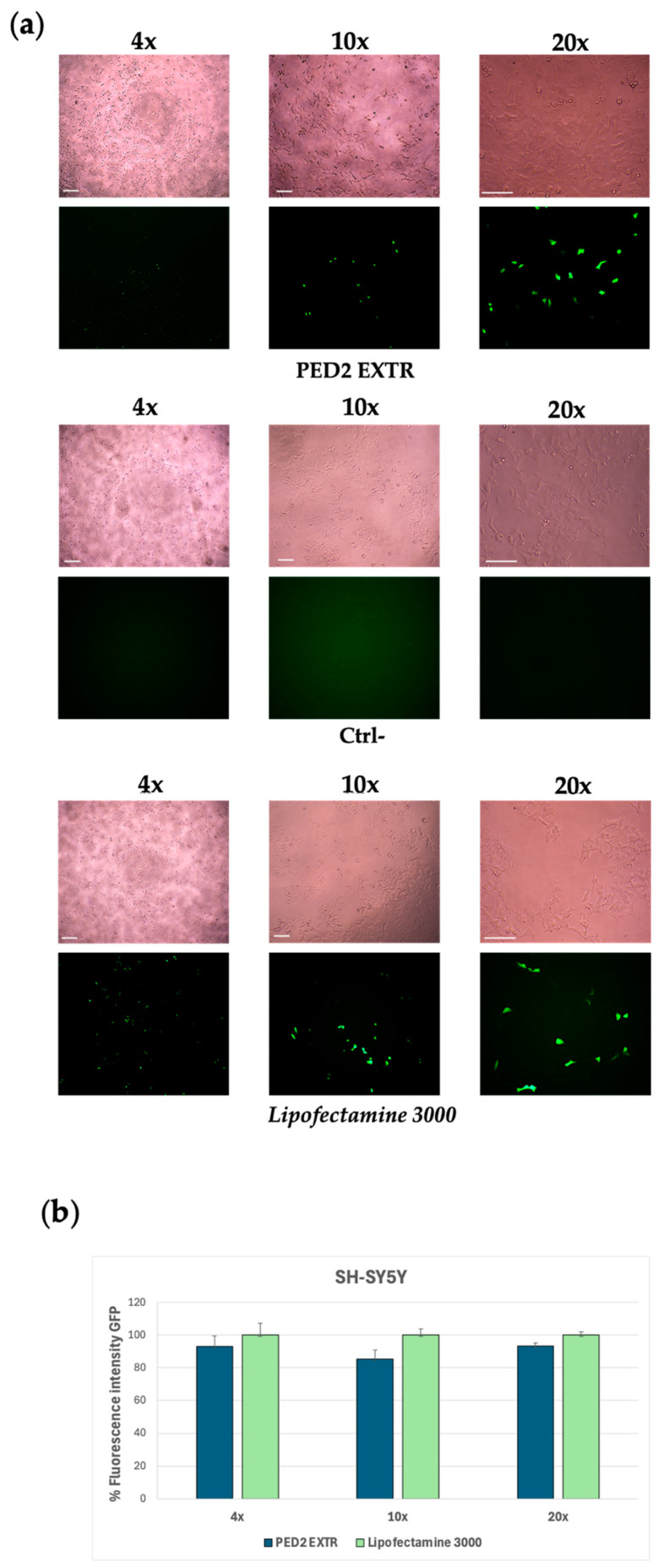
Group III formulations. (**a**) Expression of GFP in JSY-5YSH cells transfected with PED2 EXTR-eGFP liposome. The green staining represents GFP. Images were taken at 4×, 10×, and 20× magnification; scale bar is 400 µm for 4× magnification and 200 µm for 10× and 20× magnification. Lipofectamine 3000 was used as a positive control; the negative control (Ctrl−) represents cells treated with PED2 EXTR liposomes not carrying the eGFP. (**b**) Quantification of the GFP fluorescent signal in SY-5YSH cells transfected with PED2 EXTR-eGFP liposomes by ImageJ V1.53 software. Data are expressed as the mean of three different experiments + s.d.

Although a specific cytotoxicity assay (e.g., MTT) was not performed for PED2 EXTR, all transfection experiments were conducted using DDAC concentrations (10–20 µM) previously validated as non-toxic in SH-SY5Y cells (see Figure 1b for PCD2), and cell morphology and GFP expression (Figure 3b and Figure 4a) confirmed preserved viability.

Considering the encouraging results obtained with the GFP plasmid transfection experiment and the improved interaction with the pCMV-GFP plasmid, the PED2 formulation was selected to conduct further transfection studies to deliver an mRNA-expressing GFP (eGFP). SH-SY5Y cells were treated with PED2-eGFP lipoplex for 18 h, and the expression levels of GFP were then evaluated via fluorescence spectroscopy and quantified. The cells transfected with the mRNA delivered by PED2 showed a comparable fluorescence signal to the positive control cells treated with Lipofectamine 3000, indicating the efficiency of PED2 in delivering the mRNA into the cells (Figure 4a,b). Moreover, both PED1 and PED2 were monitored over time (up to day 30 post-production) in terms of dimensional stability (see Figure S1).

Although this study employed GFP-encoding plasmid and mRNA as model nucleic acids to evaluate the liposomal formulations, it is important to acknowledge the significant differences between these molecules and CRISPR/Cas9 components in terms of size, structural complexity, and delivery requirements. Specifically, CRISPR-based therapeutic approaches often involve the co-delivery of a large protein (SpCas9, ~160 kDa) or its corresponding mRNA (~4.5 kb), together with a single-guide RNA (~100 nt). These components present more demanding challenges for encapsulation, protection, and intracellular release than the smaller and structurally simpler GFP mRNA (~1 kb) or plasmid DNA (~4.7 kb) used in the present work.

Despite these differences, the liposomal formulation PED2 EXTR demonstrated favorable physicochemical properties, including high ζ-potential, dimensional stability, and efficient transfection of both DNA and mRNA, suggesting that this system possesses the flexibility to accommodate more complex payloads. The incorporation of PE and DSPE-PEG is expected to support enhanced endosomal escape and increased circulation time, both of which are essential features for the effective intracellular delivery of CRISPR components. Notably, recent studies have shown that similar cationic liposomal systems are able to mediate CRISPR/Cas9-based genome editing. For instance, Sousa et al. demonstrated in vitro gene editing using multivalent cationic liposome–DNA complexes carrying Cas9/sgRNA constructs [16], while Walther et al. compared the efficiency of lipid nanoparticle-mediated delivery of CRISPR/Cas9 as RNPs versus mRNA/sgRNA and confirmed their applicability in both in vitro and in vivo settings [17].

Based on these considerations and the promising experimental results obtained with PED2, we consider this formulation a strong candidate for future studies aimed at co-delivering Cas9 mRNA and sgRNA. Although CRISPR/Cas9 components were not directly tested in this study, the successful delivery of plasmid and mRNA cargoes by PED2 supports its potential adaptability to more complex gene editing systems. In this context, we are currently undertaking further investigations targeting disease-relevant genes such as ATXN1, with the objective of validating this approach in the treatment of spinocerebellar ataxia. In parallel, optimization of the liposomal composition, particularly regarding PEG and DSPE concentrations, is ongoing to further enhance nucleic acid encapsulation efficiency and delivery performance.

## 3. Materials and Methods

### 3.1. Materials

Phosphatidylcholine (Phospholipon 90G, PC) and pegylated 1,2-distearoyl-sn-glycero-3-phosphoethanolamine (PE 18:0/18:0-PEG 2000, DSPE-PEG2000) were purchased from Lipoid GmbH (Steinhausen, Svizzera); Phosphatidylethanolamine (PE) was purchased from Lipid Products (South Nutfield, UK); Cholesterol (CH) from Merck KGaA (Darmstadt, Germany); dioctadecyl-dimethyl-ammonium chloride (DDAC) and High-performance liquid chromatography (HPLC) grade reagents and solvents were supplied by Sigma-Aldrich (St. Louis, MO, USA). The mRNA encoding for GFP (eGFP) and Ethidium Bromide were purchased from Ribopro (Oss, The Netherlands); λDNA phage was from Pharmacia Biotech (Uppsala, Sweden). Lipofectamine™ 3000 and Opti-MEM™ were from Thermo Fisher Scientific (Waltham, MA, USA).

### 3.2. Liposomes Preparation

Liposome preparation was carried out using the ethanol injection method to obtain a final lipid concentration of 10 mg/mL. This method involves the injection of an ethanolic solution (solvent) containing lipids into an aqueous solution (non-solvent) resulting in the formation of more stable liposomes and thereby avoiding the need for sonication [51]. Initially, stock solutions of the different lipid components were prepared in ethanol, from which the proper volume of each lipid component was taken to obtain an ethanolic phase containing the different lipids at a final concentration of 10 mg/mL and at various molar ratios. For the injection of the ethanolic lipid mixture into the aqueous solution (H_2_O for HPLC), a syringe pump from Harvard Apparatus model 11 (Holliston, MA, USA) was used. Depending on the volume of ethanol, the lipid mixture was injected via insulin syringes (0.6 mL of lipid mixture in 2 mL of aqueous solution), equipped with a 26G needle, at a flow rate of 300 µL/min and 2 mL syringes (1.2 mL of lipid mixture in 4 mL of aqueous solution) at a rate of 100 µL/min, to obtain a “droplet” injection. The lipid mixture was injected into the aqueous solution under continuous stirring by using a magnetic stirrer set at 300 rpm at 25 °C. Upon injection, the formulations were left in a constant-stirring environment inside a chemical fume hood for a time varying from 3 to 4 h, until the ethanolic phase had completely evaporated. The formulations obtained were then stored at room temperature for further analysis. The ethanol injection conditions, and list of liposome formulations are reported in Table 1. For liposome extrusion, the LIPEX^®^ EXTRUDER (Lipex Biomembranes, Vancouver, BC, Canada) was used. The liposomes with average diameter higher than 200 nm, were forced to pass through a 0.2 µm pore size polycarbonate filter (Nucleopore Corp., Pleasanton, CA, USA) five times, supported by a polyester drain disk, at a pressure of 10–20 nitrogen to reduce vesicle size and obtain a homogeneous population. The liposomes were then collected and stored at room temperature for further studies.

### 3.3. Dimensional Analysis

The dimensional analysis of liposomes was performed using a Zetasizer Nano S90 (Malvern Instruments, Malvern, UK), equipped with a 5 mW He-Ne laser with a wavelength output of 633 nm, and laser angle of 90°, based on Photon Correlation Spectroscopy (PCS). In order to assess the analysis, samples were diluted 1:10 in double-distilled water up to 1 mL and transferred to transparent polystyrene cuvettes. All measurements were performed at temperature of 25 °C, in triplicate. Based on the Brownian motion of the particles, it is possible to obtain a graph displaying the mean diameter of the nanoparticle population (Z-Average) and the dimensional homogeneity of the system (Polydispersity index, PdI), whose optimal value for vesicle systems should be <0.4. Data are expressed as mean ± s.d. of triplicate determinations obtained in three independent experiments.

### 3.4. ζ-Potential Analysis

The surface charge determination of liposomes was assessed by measuring the zeta potential (ζ) using a Zetasizer PRO Nano Series (Malvern Panalytical Ltd., Malvern, UK), which follows the Electrophoretic Light Scattering (ELS) principle. For the analysis, the samples were diluted 1:10 *v*/*v* in double-distilled water and transferred to disposable capillary cells (DTS 1070, Malvern, UK). All measurements were carried out in triplicate at 25 °C. Data are expressed as mean ± SD of triplicate determinations obtained in three independent experiments.

### 3.5. Electrophoretic Run of DNA/Liposomes Complexes (Lipoplex)

The DNA/liposome complexes (lipoplexes) were prepared by co-incubating liposomes with the phage λDNA or pCMV-GFP. Precisely, 1 µg of λDNA or pCMV-GFP was added to 20 µL of liposome formulation diluted in double-distilled water to obtain final concentrations of DDAC ranging from 10 to 120 μM. The incubation was performed at room temperature for 15 min before proceeding with the agarose gel electrophoretic run to test the binding strength of lipoplexes. The agarose gel was prepared at a concentration of 0.8% *w*/*v* in a 1× TAE buffer solution (0.04 M Tris-Acetate, 0.001 M EDTA, pH 8). The solution was then boiled until the agarose powder was completely dissolved in the buffer. After cooling, the DNA intercalator ethidium bromide was added at a final concentration of 0.5 µg/mL for band detection. Subsequently, the agarose gel was poured into the electrophoretic cell to let it solidify for the electrophoretic run of the selected liposome samples. Samples were prepared by mixing 17 µL of the liposome solution at the chosen concentration of DDAC with 2 µL of a bromophenol blue solution (0.25% bromophenol blue, 30% glycerol) and 1 µL of λ DNA or pCMV-GFP. The samples were then loaded into the gel, and a voltage of 90–100 Volts was applied to induce nucleic acid migration. At the end of the run, the gel was analyzed under a UV lamp using the ChemiDocTMMP instrument (Bio-Rad, Hercules, CA, USA) to detect the fluorescent bands.

### 3.6. Cell Culture and Cytotoxicity Study (MTT)

The Neuroblastoma cell line SH-SY5Y was cultured in high glucose Dulbecco’s Modified Eagle’s Medium (EuroClone, Milan, Italy), supplemented with 15% of fetal bovine serum (FBS), 2 mM L-Glutamine, and 5 mL of an antibiotic antimycotic solution (500 U/µL of Penicillin, 0.1 µg/µL Streptomycin, 0.25 µg/µL Amphotericin B) (Merck, Darmstadt, Germany). Cells were cultured in an incubator at 37 °C in a humidified environment (5% CO_2_). The non-toxic treatment dose of liposomes was determined via an MTT (3-[4,5-dimethylthiazol-2-yl]-2,5-diphenyltetrazolium bromide) assay on SH-SY5Y, as described (ref). Briefly, 104 SH-SY5Y cells were plated in a 96-well plate in 200 µL of media and incubated at 37 °C, 5% CO_2_ for 24 h. The following day, the media was removed, and the cells were treated with 200 µL of liposomal formulations diluted in complete media at different concentrations of DDAC, ranging from 1 to 120 µM. Cells were then incubated at 37 °C in a humidified atmosphere and 5% CO_2_ for 24 h. At the end of the incubation time, the formulations were removed, and 110 µL of MTT solution (Sigma-Aldrich, St. Louis, MO, USA) at a final concentration of 0.5 mg/mL in complete media was added to each well. Cells were incubated at 37 °C in a humidified atmosphere and 5% CO_2_ for 4 h. Subsequently, the MTT solution was removed, avoiding the aspiration of the newly formed purple formazan crystal salts. Then, the crystal salts present in each well were dissolved with 100 µL of DMSO for 15 min at 37 °C in the incubator. The absorbance was measured by a spectrophotometer at a wavelength of 570 nm, using 690 nm as the reference wavelength, and then converted into % of cell viability.

### 3.7. Transfection Studies: pCMV-GFP and mRNA-GFP

For the transfection study, a plasmid able to express the green fluorescent protein GFP (pCMV-GFP) was employed. Briefly, 10^4^ cells were plated in a 96-well plate in 195 µL of supplemented media, and the plate was incubated for 24 h at 37 °C in a humidified atmosphere and 5% CO_2_. Subsequently, liposomes were filtered by using a syringe equipped with a 0.22 µm pore filter to prevent contamination. Samples were then diluted to a final concentration of DDAC of 400 µM and 800 µM, and incubated with 150 ng of pCMV-GFP for 15 min. The positive control was prepared by using Lipofectamine™ 3000. Briefly, 0.3 µL of Lipofectamine™ 3000 was added to 5 µL of Opti-MEM™ in one tube; meanwhile, 5 µL of Opti-MEM™ was mixed with 0.4 µL of P3000™ Reagent and 150 ng of pCMV-GFP in a second tube. Finally, the contents of the two tubes were combined and incubated at room temperature for 15 min. For the transfection experiment, lipoplexes were added to the cells to achieve a final concentration of DDAC of 10 µM (starting from a 400 µM liposome solution) and 20 µM (starting from an 800 µM liposome solution), in Opti-MEM. Cells treated with liposomes not carrying pCMV-GFP were used as negative control (Ctrl−). The treated cells were incubated for 5 h at 37 °C in a humidified atmosphere and 5% CO_2_. After 5 h, the transfection medium of the lipoplexes was replaced with supplemented medium, and the cells were incubated for 48 h at 37 °C in a humidified atmosphere with 5% CO_2_. The fluorescent signal was then detected and visualized by using the Eclipse Ts2 FL microscope (NIKON, Tokyo, Japan). The fluorescent signal of GFP was measured and quantified using ImageJ software (ImageJ 1.53a, Wayne Rasband, National Institutes of Health, Bethesda, MD, USA).

### 3.8. eGFP Transfection

For the transfection experiment, 10^4^ cells were plated in a 96-well plate in 195 µL of complete media, and the plate was incubated for 24 h at 37 °C in a humidified atmosphere and 5% CO_2_. Lipofectamine™ 3000 Transfection Reagent was used as a positive control. Liposomes were sterilized by filtration through 0.22 µm syringe filters and diluted in DNase-, RNase-Free H_2_O (Fisher Scientific; Hampton, EN, USA) at a concentration of 400 µM of DDAC. They were incubated for 15 min at room temperature with 100 ng of mRNA to promote complex formation. The same amount of mRNA was used to prepare the positive control as previously described. Subsequently, the culture medium was replaced with Opti-MEM™ medium, and the liposome/mRNA complexes were added to the respective wells containing SH-SY5Y. The cells were incubated for 5 h at 37 °C in a humidified atmosphere and 5% CO_2_. At the end of the incubation time, the transfection medium was removed, and the supplemented medium was added. The cells were incubated at 37 °C in a humidified atmosphere and 5% CO_2_ for 12 h. The fluorescent signal was detected and visualized using the Eclipse Ts2 FL microscope (NIKON, Tokyo, Japan) and quantified using the ImageJ software (ImageJ 1.53a, Wayne Rasband, National Institutes of Health, Bethesda, MD, USA).

### 3.9. Statistical Analysis

Statistical analysis was conducted using GraphPad Prism 9, Version 9.4.1 (GraphPad Software Inc., La Jolla, CA, USA) for all biological studies. Analysis of variance (1-way or 2-way ANOVA), followed by Tukey’s post hoc test, was used for each of the variables tested. Data are expressed as mean ± SD of duplicate determinations obtained in three independent experiments. The probability (*p*) value was considered statistically non-significant when *p* > 0.05.

## 4. Conclusions

The preliminary results of this study highlight the potential of liposomal systems as non-viral vectors for delivering nucleic acids to neuronal cells. Through a formulation screening based on physicochemical parameters, nucleic acid complexation ability, and transfection efficiency, the PED2 formulation—comprising PC:PE:DSPE-PEG:CH:DDAC in a 2:4:1:4:4 molar ratio—emerged as the most promising candidate. This cationic liposomal formulation demonstrated not only good dimensional stability and efficient complexation with nucleic acids but also successful delivery of both plasmid DNA and mRNA encoding GFP in SH-SY5Y cells, with limited cytotoxicity. These findings support the use of PED2 liposomes as a potential platform for gene therapy applications, particularly for the delivery of CRISPR/Cas9 systems targeting polyglutamine spinocerebellar ataxias and other autosomal dominant neurodegenerative diseases. Further studies are necessary to validate these findings in more complex biological models and to optimize the delivery of the CRISPR/Cas9 system for in vivo applications.

## Figures and Tables

**Table 1:** Acronyms, compositions, and preparation parameters of liposome formulations produced in the present study.

Group	Formulation Acronym	Composition	Molar Ratio (mol/mol)	Final Lipid Concentration (mg/mL)	Injection Flow Rate (µL/mL)
I	PC	PC:CH	4:1	10	300
	PCD1	PC:CH:DDAC	4.2:1	10	100
	PCD2	PC:CH:DDAC	4:2:2	10	100
II	PE1	PC:PE:CH:DDAC	3:1:2:2	10	100
	PE2	PC:PE:CH:DDAC	1:3:2:2	10	100
III	PED1	PC:PE:DSPE-PEG:CH:DDAC	1:2:1:2:2	10	100
	PED2	PC:PE:DSPE-PEG:CH:DDAC	2:4:1:4:4	10	100

PC, phosphatidylcholine; CH, cholesterol; DDAC, didodecyldimethylammonium chloride; DSPE-PEG, 1,2-distearoyl-sn-glycero-3-phosphoethanolamine-N-[methoxy(polyethylene glycol)]; PE, phosphatidylethanolamine.

**Table 2:** Dimensional parameters of Group I formulations in terms of size (Z-average), polydispersion (PdI), and surface charge properties (*ζ*-potential). Data are the mean ± SD of triplicate determinations obtained in three independent experiments.

Group I Formulation	Z-Average ± s.d. (nm)	PdI ± s.d.	ζ-Potential ± s.d. (mV)
PC	131.65 ± 1.91	0.232 ± 0.03	+26.24 ± 6.54
PCD1	54.62 ± 7.93	0.485 ± 0.04	+65.16 ± 4.25
PCD2	55.22 ± 6.90	0.432 ± 0.09	+67.75 ± 7.29

**Table 3:** Dimensional parameters of Group II formulations in terms of size (Z-average), polydispersion (PdI), and surface charge properties (*ζ*-potential). Data are expressed as mean ± SD of triplicate determinations obtained in three independent experiments.

Group II Formulation	Z-Average ± s.d. (nm)	PdI ± s.d.	ζ-Potential ± s.d. (mV)
PE1	341.85 ± 13.08	0.570 ± 0.08	+62.01 ± 2.72
PE1 EXTR	203.35 ± 0.08	0.129 ± 0.03	+59.14 ± 1.23
PE2	145.57 ± 3.58	0.172 ± 0.01	+91.07 ± 4.65

**Table 4:** Dimensional parameters of Group III formulations in terms of size (Z-average), polydispersion (PdI), and surface charge properties (*ζ*-potential). Data are expressed as mean ± SD of triplicate determinations obtained in three independent experiments.

Group III Formulation	Z-Average ± s.d. (nm)	PdI ± s.d.	ζ-Potential ± s.d. (mV)
PED1	709.70 ± 300.04	0.219 ± 0.09	+62.67 ± 3.48
PED1-EXTR	149.85 ± 37.11	0.169 ± 0.01	+74.66 ± 2.16
PED2	1101.00 ± 428.99	0.290 ± 0.05	+48.76 ± 1.53
PED2-EXTR	106.83 ± 9.12	0.368 ± 0.05	+53.99 ± 1.74

## Data Availability

Data is contained within the article or Supplementary Materials.

## Supplementary Materials

The following supporting information can be downloaded at: https://www.mdpi.com/article/10.3390/molecules30173585/s1, Table S1. Composition and N/P ratio of the formulations used for the complexation studies. Figure S1. Dimensional stability of PED formulations over time before extrusion expressed as mean diameters (Z-average) and polydispersity index values (PdI).

## Abbreviations

The following abbreviations are used in this manuscript:

CH	Cholesterol
DDAC	Dioctadecyl-dimethylammonium chloride
DSPE-PEG	Pegylated 1,2-distearoyl-sn-glycero-3-phosphoethanolamine
PC	Phosphatidylcholine
GFP	Green fluorescent protein
PE	Phosphatidylethanolamine
SCAs	Spinocerebellar ataxia disorders
