# Supplementary material for: A Pre-Formulation Study for Delivering Nucleic Acids as a Possible Gene Therapy Approach for Spinocerebellar Ataxia Disorders
Source: Molecules. 2025 Sep 2;30(17):3585. doi: 10.3390/molecules30173585 (PMC12430385; doi:10.3390/molecules30173585)
Supplement: Supplementary file 1 [file molecules-30-03585-s001.zip › molecules-3782875-supplementary.pdf]

Supplementary Material

A pre-formulation study for delivering nucleic acids as a possible gene therapy approach for spinocerebellar ataxia disorders

Francesca Ferrara, Alfredo Sepe, Maddalena Sguizzato, Peggy Marconi and Rita Cortesi*

Table S1. Composition and N/P ratio of the formulations used for the complexation studies.

Group	Formulation acronym	Composition	Molar ratio (mol/mol)	Nucleic acid cargo type (1 μg)	DDAC (μM)	N/P ratio
I	PCD2	PC:CH:DDAC	4:2:2	λDNA	120	2.4/1
					80	1.6/1
					60	1.2/1
					40	0.8/1
II	PE1	PC:PE:CH:DDAC	3:1:2:2	pCMV-GFP	20	40/1
					10	20/1
	PE2	PC:PE:CH:DDAC	1:3:2:2	pCMV-GFP	20	40/1
					10	20/1
III	PED1	PC:PE:DSPE-PEG:CH:DDAC	1:2:1:2:2	pCMV-GFP	20	40/1
					10	20/1
	PED2	PC:PE:DSPE-PEG:CH:DDAC	2:4:1:4:4	pCMV-GFP	20	36/1
					10	18/1

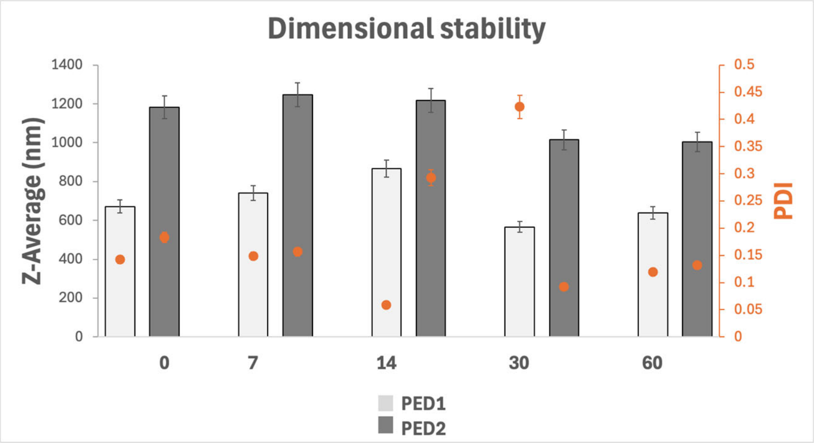

Figure S1. Dimensional stability of PED formulations over time before extrusion expressed as mean diameters (Z-average) and polydispersity index values (PdI).
